# A computational model-based study on the feasibility of predicting post-splenectomy thrombosis using hemodynamic metrics

**DOI:** 10.3389/fbioe.2023.1276999

**Published:** 2024-01-11

**Authors:** Tianqi Wang, Yan Yong, Xinyang Ge, Jitao Wang

**Affiliations:** ^1^ School of Gongli Hospital Medical Technology, University of Shanghai for Science and Technology, Shanghai, China; ^2^ School of Mechanical Engineering, University of Shanghai for Science and Technology, Shanghai, China; ^3^ College of Science, University of Shanghai for Science and Technology, Shanghai, China; ^4^ College of Mathematical Medicine, Zhejiang Normal University, Jinhua, China; ^5^ Department of Hepatobiliary Surgery, Xingtai Institute of Cancer Control, Xingtai, China

**Keywords:** splenectomy, post-splenectomy thrombosis, hemodynamics, computational model, wall shear stress

## Abstract

For portal hypertensive patients with splenomegaly and hypersplenism, splenectomy is an effective surgery to relieve the complications. However, patients who have undergone splenectomy often suffer from portal venous system thrombosis, a sequela that requires prophylaxis and timely treatment to avoid deterioration and death. The aim of this study is to investigate the feasibility of predicting post-splenectomy thrombosis using hemodynamic metrics based on computational models. First, 15 portal hypertensive patients who had undergone splenectomy were enrolled, and their preoperative clinical data and postoperative follow-up results were collected. Next, computational models of the portal venous system were constructed based on the preoperative computed tomography angiography images and ultrasound-measured flow velocities. On this basis, splenectomy was mimicked and the postoperative area of low wall shear stress (ALWSS) was simulated for each patient-specific model. Finally, model-simulated ALWSS was statistically compared with the patient follow-up results to investigate the feasibility of predicting post-splenectomy thrombosis using hemodynamic metrics. Results showed that ALWSS could predict the occurrence of post-splenectomy thrombosis with the area under the receiver operating characteristic curve (AUC) equal to 0.75. Moreover, statistical analysis implied that the diameter of the splenic vein is positively correlated with ALWSS (*r* = 0.883, *p* < 0.0001), and the anatomical structures of the portal venous system also influence the ALWSS. These findings demonstrated that the computational model-based hemodynamic metric ALWSS, which is associated with the anatomorphological features of the portal venous system, is capable of predicting the occurrence of post-splenectomy thrombosis, promoting better prophylaxis and postoperative management for portal hypertensive patients receiving splenectomy.

## 1 Introduction

Portal hypertension is a clinical syndrome caused by elevated blood pressure in the portal venous system. Continuous progression in portal hypertension may present symptoms such as gastric hemorrhage and splenomegaly, which could be life-threatening. In the treatment of portal hypertension, splenectomy implemented in combination with porta-azygous devascularization is an effective surgery for portal hypertensive patients with splenomegaly and hypersplenism. On the other hand, splenectomy is often accompanied by a significantly increased risk of portal venous system thrombosis (Ushitora et al., 2011; Qi et al., 2016). In order to avoid intestinal ischemia and infarction or other severe complications caused by post-splenectomy thrombosis, appropriate prophylaxis or timely treatment after splenectomy is necessary (Parikh et al., 2010; Ruiz-Tovar and Priego, 2016). However, due to the lack of an effective risk prediction scheme for post-splenectomy thrombosis for portal hypertensive patients, anticoagulant therapy and postoperative management after splenectomy still need improvement. Some clinical studies investigating the risk factors of post-splenectomy thrombosis indicated that the diameter of the splenic vein and the diameter of and flow velocity in the portal vein are associated with the risk of post-splenectomy thrombosis (Danno et al., 2009; de’Angelis et al., 2017; Huang et al., 2018). Nevertheless, hemodynamic metrics, which are more direct and proved to be related to thrombosis, were not considered in those studies. Previous studies revealed that long-term exposure to an abnormal hemodynamic environment, like low wall shear stress, can induce endothelial dysfunction and thus increase the risk of venous thrombosis (Esmon, 2009; Poredos and Jezovnik, 2018). Given that hemodynamic metrics in the portal venous system will change considerably after splenectomy, it is reasonable to hypothesize that hemodynamic factors might play some roles in the initiation of post-splenectomy thrombosis. However, direct measurements of hemodynamic metrics under *in vivo* conditions, especially the details of wall shear stress, are challenging due to the limitations of available equipment.

In this context, computational models may provide a feasible approach. Some previous studies employed computational models to address various issues related to the portal venous system such as the diagnosis of portal hypertension (Wang et al., 2017; Wang et al., 2018; Wang et al., 2020) and the hemodynamic changes due to cirrhosis (George et al., 2015; Wei et al., 2017). In our previous study, the influence of anatomorphological features of the portal venous system on post-splenectomy hemodynamic characteristics was investigated based on idealized computational models (Wang et al., 2021). Moreover, we also conducted a proof-of-concept study that preliminarily indicated the association between hemodynamic metrics and post-splenectomy thrombosis based on three patient-specific models (Wang et al., 2022). The purpose of the present study was, therefore, to apply computational models to further prove the feasibility of predicting post-splenectomy thrombosis using hemodynamic metrics. For this purpose, the preoperative clinical data of 15 portal hypertensive patients were collected, based on which the patient-specific models were constructed. Subsequently, splenectomy was mimicked, and the distributions of wall shear stress were simulated. On this basis, the area of low wall shear stress was statistically compared with the status of post-splenectomy thrombosis monitored during patient follow-up to analyze the feasibility of predicting post-splenectomy thrombosis using hemodynamic metrics.

## 2 Methods

**FIGURE 1:**
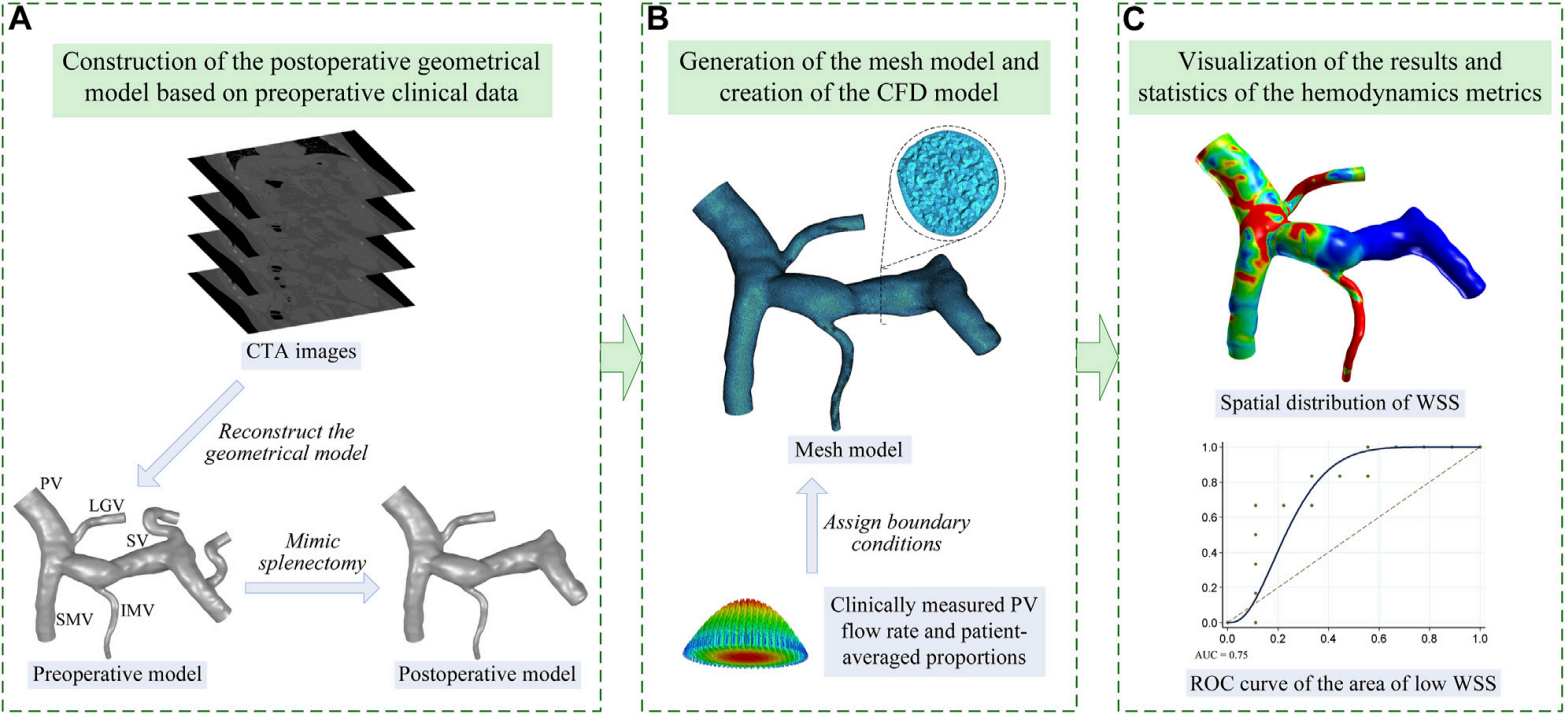
Workflow of the present study which is divided into three steps: **(A)** step 1, image-based construction of the geometrical model; **(B)** step 2, generation of the mesh model and creation of the computational fluid dynamic (CFD) model; **(C)** step 3, visualization and statistical analysis of the model-simulated wall shear stress (WSS). The name of each vein is marked in panel **(A)** including the PV, SMV, SV, LGV, and IMV.

Figure 1 shows the workflow of the present study, including 1) construction of the geometrical model based on the clinical data, 2) generation of the mesh model and creation of the computational model, and 3) statistical analysis of the simulated hemodynamic metrics. In order to clarify the technical details of this workflow, the methods of clinical data acquisition, model construction, and statistical analysis are introduced in this section.

### 2.1 Clinical data acquisition

In total, 15 portal hypertensive patients (five males and ten females, age: 54 ± 11) treated with splenectomy were retrospectively included in this study upon approval by the institution’s ethics committee (Xingtai People’s Hospital, grant number: 2022006). In the present study, all the patients were randomly selected from the database in line with the following inclusion criteria: 1) patients had decompensated cirrhosis with similar complications including splenomegaly and hypersplenism; 2) patients did not have thrombophilia or other blood disorders that might significantly increase the risk of thrombosis; 3) the quality of medical images should be good enough for geometrical model reconstruction; 4) the flow velocity in the portal vein should be available; 5) the postoperative follow-up examination should be implemented normally. While we excluded the patients whose blood constituents showed significant abnormalities, the quality of clinical data was not suitable for constructing computational models, or postoperative follow-up results were missing. Before the surgery of each patient, abdominal computed tomography angiography (CTA) scanning was performed using advanced CT equipment with small slice thickness, and the flow velocity in the portal vein was measured with a Doppler ultrasound device. During the surgery, the spleen was removed, and the residual splenic vein was ligated near the splenic hilum. Three months after each surgery, the patient was examined with an abdominal CTA to screen for potential post-splenectomy thrombosis. The follow-up results indicated that 40% (6/15) of the patients suffered from post-splenectomy thrombosis. The preoperative CTA images, ultrasound-measured flow velocity in the portal vein, and the status of post-splenectomy thrombosis monitored during the follow-up of each patient were collected for the model construction and statistical analysis in the present study. Moreover, the aforementioned clinical data of another three patients with similar conditions from Shanghai Jiao Tong University Affiliated Sixth People’s Hospital were also collected for the external validation of the obtained results.

### 2.2 Model construction

#### 2.2.1 Image-based construction of the geometrical model and generation of the mesh model

The CTA images acquired from each patient were segmented using the well-validated commercial package Mimics (Materialise, Belgium) to reconstruct a preoperative geometrical model of the portal venous system including the portal vein (PV), superior mesenteric vein (SMV), splenic vein (SV), left gastric vein (LGV), and inferior mesenteric vein (IMV), as is shown in Figure 1A. Based on the preoperative model, the proximal ends of the SV were manually closed (by means of moving the triangular facets on the vascular wall to form a closed end) to mimic the ligation treatment for the residual SV in splenectomy, while the rest of the preoperative model remained, thereby generating a postoperative geometrical model, as is shown in Figure 1A. Herein, it was assumed that splenectomy would not considerably alter the morphological features of the rest of the portal venous system. The rationality of this assumption was proven by comparing one preoperative geometrical model and the corresponding follow-up image-based postoperative geometrical model. Please see the Supplementary Material for more details.

Mesh models were generated based on the postoperative geometrical models using ICEM CFD (ANSYS Inc., United States) with a hybrid meshing strategy, where the meshing of the entire fluid domain was first implemented using tetrahedral elements, followed by a mesh refinement treatment for the near-wall region by mapping five prism layers along the vascular walls, as is shown in Figure 1B. The minimum size of tetrahedral elements was set to 0.08 mm, and the thickness of the first near-wall prism layer was set to 0.05 mm, which was increased by a ratio of 1.2 toward the internal layers. The grid convergence test performed on the model showed that further reducing the minimum size of tetrahedral elements or the thickness of the first prism layer induced negligible changes in computed hemodynamic metrics. Therefore, the aforementioned element sizes were applied to all models. Each mesh model contained approximately 1.1 million to 2.7 million elements depending on the size of the corresponding geometrical model.

#### 2.2.2 Boundary conditions and setup of the computational model

The distal end of the PV was set as the pressure outlet boundary, with the blood pressure fixed at 25 mmHg, a common value in patients with portal hypertension (Lebrec et al., 1997). Meanwhile, according to the physiological condition, the proximal ends of the SMV, SV, LGV, and IMV were set as inflow boundaries. Each of the inflow boundaries was defined in the form of a flow velocity inlet, where a cross-sectional mean flow velocity was imposed by assuming a parabolic velocity profile, as is shown in Figure 1B. The volumetric flow rate in the PV (i.e., the sum of the flow rates in the SMV, SV, LGV, and IMV) could be calculated based on the ultrasound-measured flow velocity and the diameter measured in the geometrical model. On this basis, the volumetric flow rates in the SMV, SV, LGV, and IMV could be assigned according to the population-averaged proportions of the flow rates of the portal venous system of portal hypertensive patients (Wang et al., 2021). Accordingly, the flow velocity in each vein could be calculated based on the assigned volumetric flow rate and the diameter measured in the geometrical model. Given the weak pulsation of blood flow in the portal venous system, all flow velocities imposed at the model inlets were assumed to be constant, which is consistent with the assumptions adopted in previous studies (Ho et al., 2013; George et al., 2015; Wei et al., 2017). The portal venous walls were assumed to be rigid, to which the no-slip boundary condition was imposed.

In the computational model, blood was modeled as an incompressible non-Newtonian fluid and blood flow was governed by the continuity and Navier–Stokes equations. The non-Newtonian rheology of blood was herein considered because blood flows slowly in the portal venous system, especially after splenectomy, and under such conditions, the shear-rate-dependent change in blood viscosity would become evident (Ho et al., 2012). In this study, the Carreau model was employed to represent the change in blood viscosity with shear rate (Johnston et al., 2004). The governing equations of blood flow were numerically solved using Fluent (ANSYS Inc., United States). Although the boundary conditions were prescribed using constant values, herein transient numerical schemes were adopted for the purpose of improving the numerical stability. Each set of numerical simulations for blood flow was continuously run for 3 seconds (with the time step being fixed at 0.01 s) to dissipate the errors in the estimated initial conditions and reach a converged numerical solution, and, accordingly, the numerical results obtained at the last time step were analyzed to derive hemodynamic metrics of interest.

### 2.3 Statistical analysis

In the present study, wall shear stress was selected to predict the risk of post-splenectomy thrombosis, considering the close association of low wall shear stress with blood flow stasis, endothelial cell dysfunction, and venous thrombosis (Bergan et al., 2006; Moustafa et al., 2018; Poredos and Jezovnik, 2018). Although the commonly used hemodynamic metrics to identify the risk of thrombosis include not only wall shear stress but also shear strain rate, oscillatory shear index, residence time, and stagnation index, according to previous studies (Gorring et al., 2015), most of them were used for the pulsatile flow, where the flow velocity varied remarkably and periodically. For hemodynamic studies on the portal venous system, where constant inflows were usually adopted, wall shear stress was always used to evaluate the risk of portal venous system thrombosis (George et al., 2015; Wei et al., 2017). Accordingly, in the present study, we only employed wall shear stress to predict the risk of post-splenectomy thrombosis in order to make the study more focused and be consistent with previous studies. It is assumed that with the increase in the area of low wall shear stress (ALWSS), the probability of the occurrence of post-splenectomy thrombosis would increase. Herein, ALWSS was defined as the area of the portal venous system exposed to wall shear stress lower than the threshold value, which is a patient-specific value set as 20% of the space-averaged wall shear stress of the portal venous system. After the simulation of each computational model, the distribution of wall shear stress was obtained and the space-averaged wall shear stress and ALWSS were calculated accordingly. The computed ALWSSes, in combination with the status of post-splenectomy thrombosis monitored during patient follow-ups, were employed to draw the receiver operating characteristic (ROC) curve, as is shown in Figure 1C. On this basis, the area under the ROC curve (AUC) was calculated to estimate the efficiency of predicting post-splenectomy thrombosis using ALWSS, and the feasibility of this scheme was also examined using the external validation data, which contained the data of three patients from another medical center. Furthermore, the correlation analyses were conducted to analyze the correlation between ALWSS and the velocity in the PV or the diameter of the SV to reveal the factors affecting ALWSS. Moreover, the association of ALWSS with the types of anatomical structures of the portal venous system was also investigated. According to the previous studies (Sakaguchi et al., 2010; Khamanarong et al., 2016), the anatomical structures of the portal venous system included in the present study could be divided into three types based on the connecting position of the LGV and IMV. Herein, Type 1 represents the LGV connected with the SV and IMV connected with the SV; Type 2 represents the LGV connected with the PV and IMV connected with the SV; Type 3 represents the LGV connected with the SV and IMV connected with the SMV.

## 3 Results

### 3.1 Comparison of clinical data among patients

**FIGURE 2:**
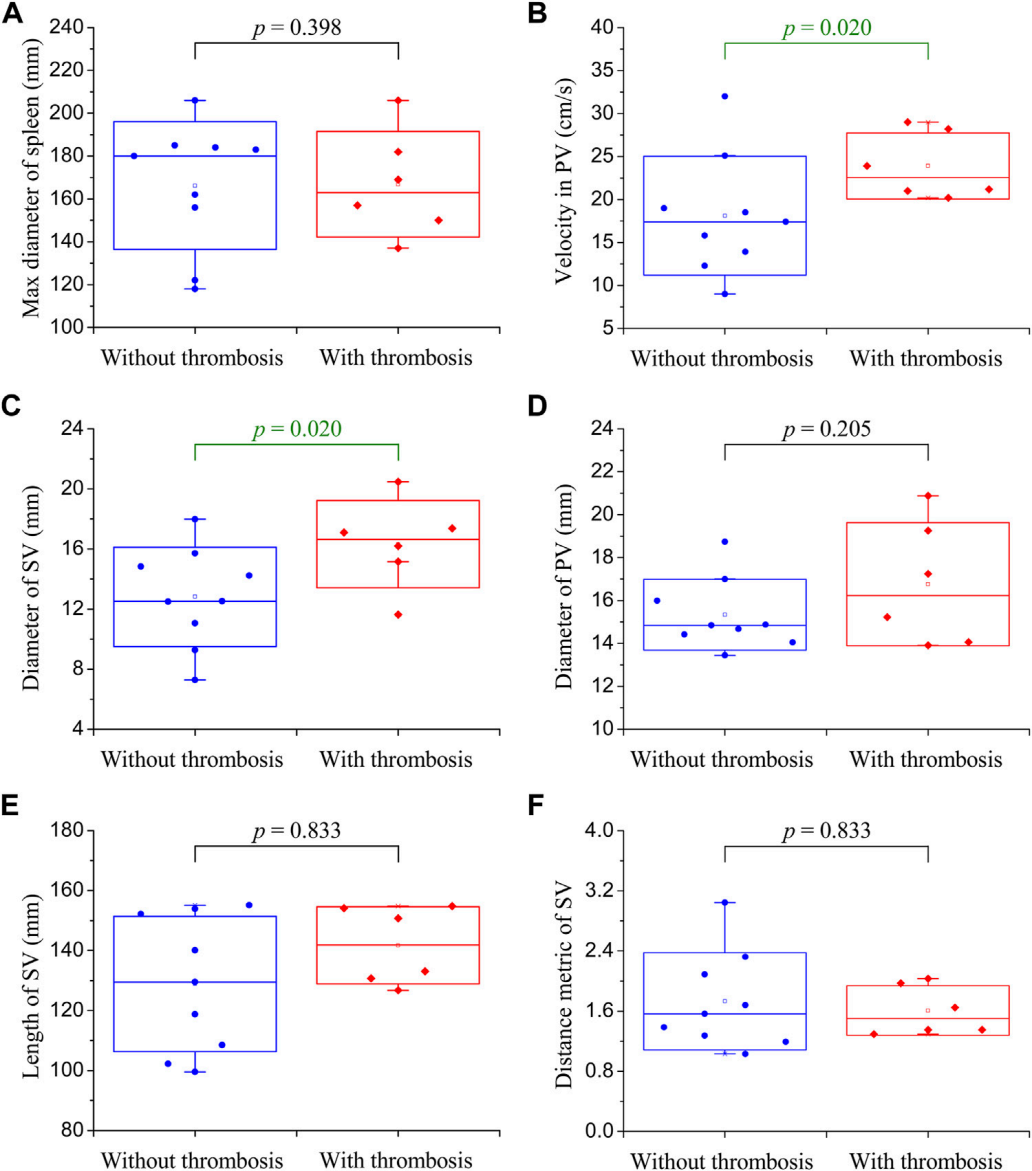
Box plots of the clinical data of enrolled patients. The small square in the box denotes the mean value, the upper and lower edges of the box represent the mean value ± standard deviation (SD), and the statistical significance of the difference between the blue group (without post-splenectomy thrombosis) and red group (with post-splenectomy thrombosis) plotted in each panel is evaluated with the *p*-value. **(A)** Maximum diameter of the spleen; **(B)** velocity in the PV; **(C)** diameter of the SV; **(D)** diameter of the PV; **(E)** length of the SV; **(F)** distance metric of the SV.

Figure 2 shows the clinical data collected from the 15 enrolled patients. Each set of data was divided into two groups according to the status of post-splenectomy thrombosis monitored during follow-ups (i.e., the blue group and red group in Figure 2). Moreover, the difference between the two groups was compared using Mood’s Median Test, and the statistical significance of the difference (evaluated with the *p*-value) is listed in each panel. It was found that the maximum diameters of the spleens were larger than the normal size, indicating that the enrolled patients suffered from splenomegaly (Figure 2A). The velocity in the PV and the diameter of the SV were the two factors that showed a statistically significant difference (*p* < 0.05) between patients free from and those suffering from post-splenectomy thrombosis (Figure 2B,C). Other factors such as the diameter of the PV, length of the SV, and distance metric of the SV were not significantly different between the two groups, although they showed wide distribution ranges (Figures 2D,F). These clinical data, on the one hand, described the characteristics of the enrolled patients; on the other hand, they implied that the velocity in the PV and the diameter of the SV were the two potential factors that might influence the occurrence of post-splenectomy thrombosis.

### 3.2 Comparison of model-simulated hemodynamic metrics

**FIGURE 3:**
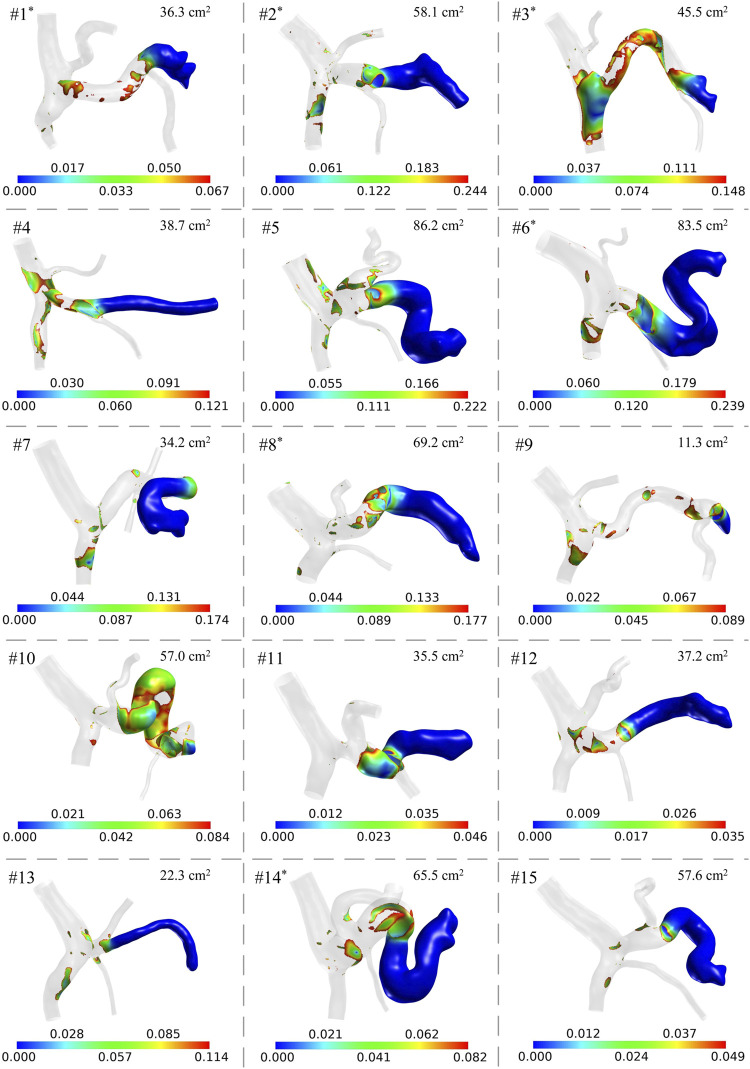
Model-simulated spatial distributions of low wall shear stress (unit: Pa) in the 15 patients. The gray region has wall shear stress higher than the threshold value of each model. ALWSS is shown in the top right corner of each panel. The superscript ‘*’ on the patient number indicates that post-splenectomy thrombosis was detected according to the follow-up results of the patient.

The geometrical models together with the spatial distributions of low wall shear are shown in Figure 3. It can be observed that the anatomical structures and morphological features varied considerably among the patients. For example, in some patients, the SVs were slender and straight, while in other patients, the SVs were wide and curved, which is consistent with the distributions of clinical data shown in Figures 2C,E,F. The colorful region shown in each panel is the venous wall covered with low wall shear stress, and the upper limit is 20% of the space-averaged wall shear stress of the corresponding model, while the gray region represents the venous wall covered with wall shear stress higher than the threshold value. It can be found that the threshold value differed from one patient to another, while they were of similar orders of magnitude, ranging from 0.035 Pa to 0.239 Pa (0.125 ± 0.069 Pa). The venous wall covered with low wall shear stress is mainly located on the SV and the junction of the SV, SMV, and PV. The area of the colorful region is the ALWSS defined in the present study, which is listed in the top right corner of each panel. In this patient cohort, ALWSS showed a marked variation from 11.3 cm^2^ to 86.2 cm^2^, indicating highly specific hemodynamic conditions of the portal venous systems of the enrolled patients after splenectomy.

### 3.3 Statistical analysis results

**FIGURE 4:**
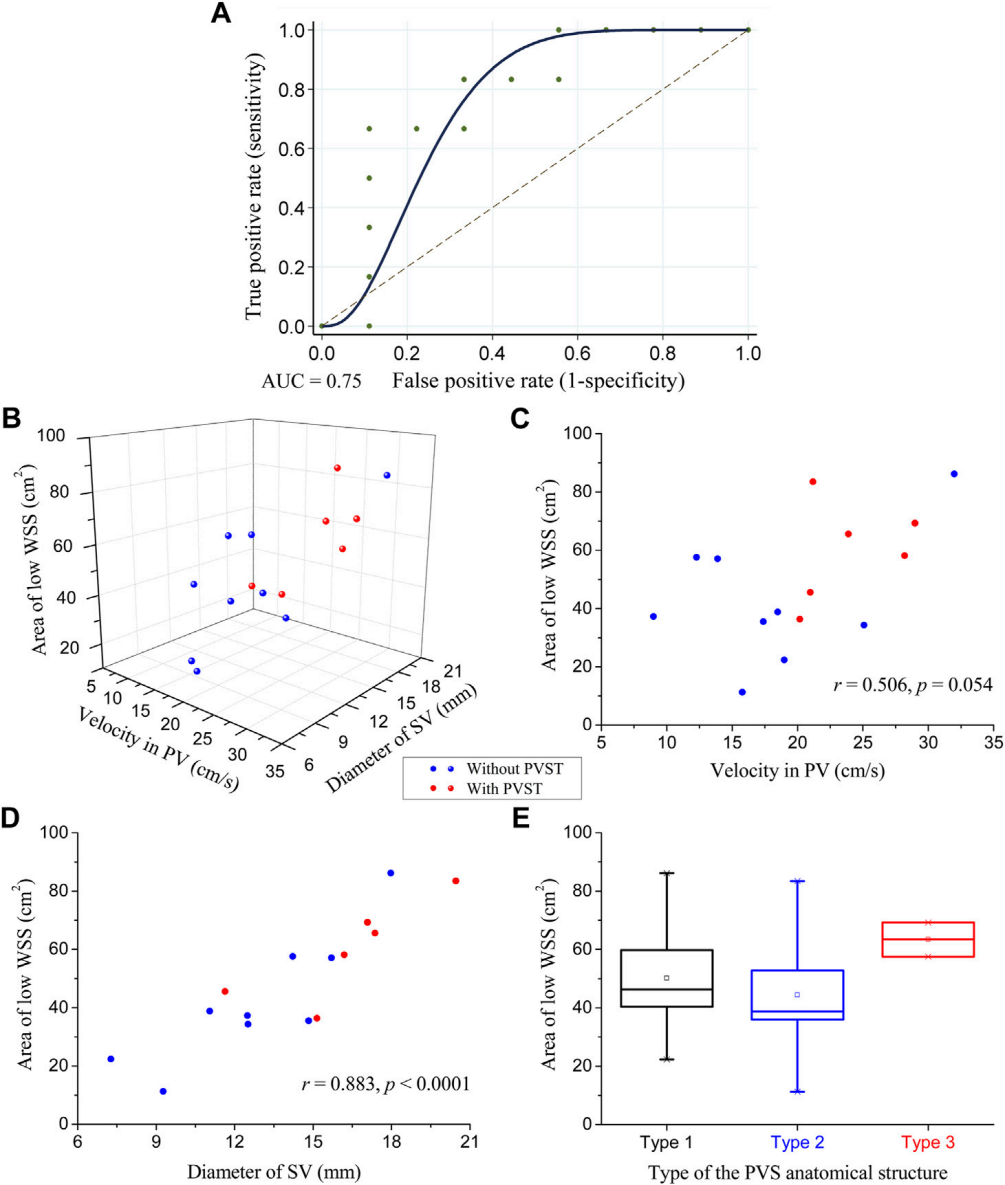
Statistical analysis results of ALWSS: **(A)** receiver operating characteristic curve of ALWSS for predicting the occurrence of post-splenectomy thrombosis with an AUC equal to 0.75; **(B)** three-dimensional scatter plot of ALWSS against the velocity in the PV and the diameter of the SV; **(C)** two-dimensional scatter plot of ALWSS against the velocity in the PV and their correlation analysis results; **(D)** two-dimensional scatter plot of ALWSS against the diameter of the SV and their correlation analysis results; **(E)** box plots of ALWSS in the three patient groups with three different types of anatomical structures of the portal venous system. The small square in the box denotes the mean value, and the upper and lower edges of the box represent the mean value ± standard error (SE). The red/blue balls in panels **(B)**, **(C)**, and **(D)** represent the patients with/without post-splenectomy thrombosis.

The ROC curve of the ALWSS for predicting post-splenectomy thrombosis is shown in Figure 4A, representing the variation of the true positive rate (i.e., sensitivity) against the false positive rate (i.e*.*, 1—specificity) when using different threshold values of ALWSS to predict the occurrence of post-splenectomy thrombosis. AUC equaled 0.75, and the correctly classified ratio reached 80% when choosing 58.1 cm^2^ as the threshold value of ALWSS (i.e., judging post-splenectomy thrombosis would occur if the ALWSS was higher than this value). External validation using the clinical data of three patients from another medical center indicated that the ALWSS with this threshold value could be used to identify the patients with/without post-splenectomy thrombosis correctly. Please see the Supplementary Material for the details of the results of the external validation. These results implied that ALWSS could be an effective hemodynamic metric for predicting the occurrence of post-splenectomy thrombosis. Further analysis of the correlation between ALWSS and the two factors showing statistically significant difference between patients free from and those suffering from post-splenectomy thrombosis revealed that ALWSS was significantly positively correlated with the diameter of the SV, with a Pearson correlation coefficient equal to 0.883 (*p* < 0.0001), while ALWSS was not statistically significantly correlated with the velocity in the PV (*p* = 0.054) (Figures 4B–D). Moreover, Figure 4E shows the box plots of ALWSS in the three patient groups with three different types of anatomical structures of the portal venous system, namely, Type 1, Type 2, and Type 3, as defined in Subsection 2.3. It can be observed that ALWSS was higher in the Type 3 group than in the other two groups, demonstrating that ALWSS was also influenced by the anatomical structures of the portal venous system.

## 4 Discussion

Post-splenectomy thrombosis is a thorny sequela for portal hypertensive patients after splenectomy. Due to the lack of an effective risk prediction scheme for the occurrence of post-splenectomy thrombosis, patients usually receive uniform postoperative management including the same anticoagulant therapy and the same follow-up CTA scanning arrangement, leading to the ineffective prophylaxis and untimely detection of post-splenectomy thrombosis in some high-risk patients. Although previous clinical studies revealed the influence of venous diameters or flow velocity on the risk of post-splenectomy thrombosis, they did not investigate the influence of hemodynamic metrics which are more essential.

In the present study, 15 patients were included and the patient-specific computational models were constructed to simulate the distribution of wall shear stress and calculate ALWSS. Data analysis results demonstrated the feasibility of predicting post-splenectomy thrombosis using the hemodynamic metric ALWSS with an AUC equal to 0.75. Meanwhile, this AUC value also implied that other factors might also influence the formation of post-splenectomy thrombosis. In previous studies, Virchow famously postulated three main causes of thrombosis, namely, stasis of the blood, changes in the vascular wall, and changes in the composition of the blood (Rosendaal, 1993; Esmon, 2009). While wall shear stress can reflect the conditions of blood flow stasis and endothelial cell dysfunction, it cannot reflect the blood constituents like protein C and protein S, which may play a role in portal venous system thrombosis (Sobhonslidsuk and Reddy, 2002). In the present study, we excluded patients with blood diseases when collecting clinical data; however, it is very hard to ensure the blood constituents of the enrolled patients were all the same. This may be why the hemodynamic metric ALWSS cannot totally describe the formation of a thrombus. Nevertheless, this AUC value still indicated that ALWSS has acceptable efficiency in predicting post-splenectomy thrombosis.

Considering that hemodynamic metrics are usually influenced by the blood flow status in the portal venous system, the geometry of the fluid domain would remarkably influence the results. The analysis results did confirm this opinion, disclosing the association between ALWSS and the diameter of SV or the type of the anatomical structure of the portal venous system. These results, on the one hand, provided a feasible approach for predicting the occurrence of post-splenectomy thrombosis in clinical practice; on the other hand, they revealed the underlying anatomorphological factors of the portal venous system influencing ALWSS.

These findings suggest that conducting hemodynamic analysis before splenectomy would assist in identifying high-risk patients and thus improve postoperative management. Taking the results of computational model-based hemodynamic analysis into account when arranging postoperative management for portal hypertensive patients after splenectomy, patient-specific anticoagulant prophylaxis and a more reasonable follow-up CTA scanning frequency would be adopted, which would improve the outcome of splenectomy for portal hypertensive patients with splenomegaly and hypersplenism. The advantage of this risk prediction scheme also includes that the clinical data used to construct the patient-specific computational models were all collected during routine examinations before splenectomy, which would not bring any additional burden to the patients. This advantage would also make the clinical trials and practical applications of this scheme more feasible in the future.

Admittedly, this study has certain limitations. The number of enrolled patients still needs to be increased in order to get a more accurate threshold value of ALWSS for judging the occurrence of post-splenectomy thrombosis. Nevertheless, the tendency disclosed by the present study is still robust enough to support the feasibility of predicting post-splenectomy thrombosis using hemodynamic metrics. Furthermore, the influence of blood constituents was not considered since the enrolled patients in the present study were under similar conditions without blood diseases and the difference between their blood constituents was not remarkable. In future studies, blood constituents and blood disorders like thrombophilia that might influence the occurrence of venous thrombosis may be investigated together with the hemodynamic metrics to establish a more robust and mixed predictive model and provide a more general insight for the prediction of post-splenectomy thrombosis.

## Data Availability

The raw data supporting the conclusion of this article will be made available by the authors, without undue reservation.
