# Supplementary material for: A computational model-based study on the feasibility of predicting post-splenectomy thrombosis using hemodynamic metrics
Source: Front Bioeng Biotechnol. 2024 Jan 11;11:1276999. doi: 10.3389/fbioe.2023.1276999 (PMC10808826; doi:10.3389/fbioe.2023.1276999)
Supplement: Supplementary file 1 [file DataSheet1.PDF]

Supplementary Material

A computational model-based study on the feasibility of predicting postsplenectomy thrombosis using hemodynamic metrics

Tianqi Wang[1, 2], Yan Yong[3], Xinyang Ge[4], Jitao Wang[5*]

*** Correspondence:** Jitao Wang: wangjt302@163.com

1 Supplementary Data

External validation was conducted to examine the feasibility of predicting postsplenectomy thrombosis using ALWSS with the threshold value obtained from the receiver operating characteristic analysis. Supplementary Figure 1 shows the results of the three patients for external validation. ALWSS is shown in the top right corner of each panel where blue/red represents that the value is lower/higher than the threshold value of ALWSS (58.1 cm^2) in the present study. The superscript '*' on the patient symbol indicates that postsplenectomy thrombosis was detected according to the follow-up results of the patient. It can be found that Patient A and Patient B were free from postsplenectomy thrombosis and their ALWSSes were 22.2 cm^2 and 54.6 cm^2, respectively, which were both lower than the threshold value. While Patient C was the patient suffering from postsplenectomy thrombosis, and the corresponding ALWSS reached 63.4 cm^2 which was higher than the threshold value. These results indicated that ALWSS could be used to identify the patients with/without postsplenectomy thrombosis correctly.

In the present study, it was assumed that splenectomy would not considerably alter the morphological features of the rest of the portal venous system. In order to prove that this assumption is reasonable, we reconstructed a geometrical model based on the CTA images acquired during postoperative follow-up (the third month) of Patient C and compared it with the preoperative one (Supplementary Figure 2). Herein, the range of postoperative model reconstruction was confined to the region near the junction of PV, SV and SMV because other regions were hard to be reconstructed due to low contrast ratio, small vascular diameter, and the interference of thrombus. It is noted that the postoperative model is rough and hard to be smoothed because the quality of the follow-up medical images is low and the vascular lumen is partly occupied by thrombus. Nevertheless, the outlines of the preoperative and postoperative models are highly similar, which provides evidence for our assumption.

2 Supplementary Figures

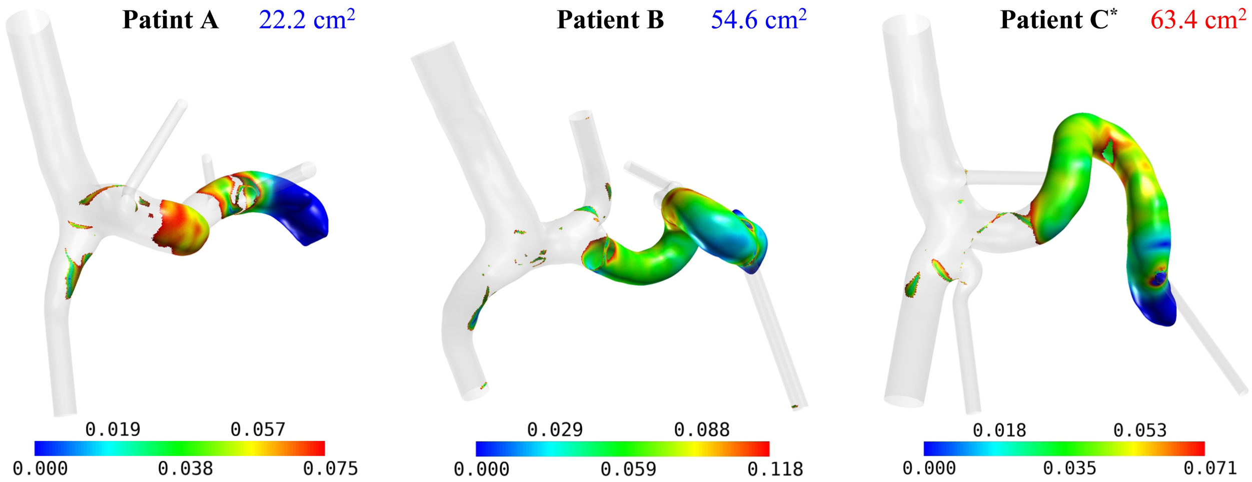

Supplementary Figure 1. Model-simulated spatial distributions of low wall shear stress (unit: Pa) of the three patients for external validation.

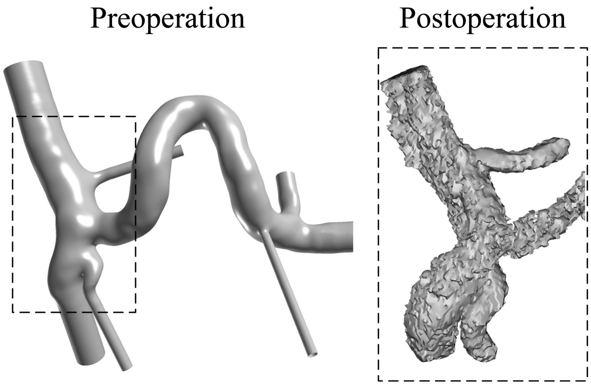

Supplementary Figure 2. Geometrical models of the portal venous system reconstructed based on CTA images taken before and after splenectomy in Patient C.
